# Supplementary material for: An automatic water-occluding device to enable laryngectomee participation in water activities
Source: PLoS One. 2021 Sep 13;16(9):e0257463. doi: 10.1371/journal.pone.0257463 (PMC8437266; doi:10.1371/journal.pone.0257463)
Supplement: S1 File — Survey administered to the study participants. (PDF) [file pone.0257463.s001.pdf]

1. How long has it been since your laryngectomy?
a) Less than 6 months
b) 6 months to a year
c) 1 year to 2 years
d) Greater than 2 years

2. What form of speech do you prefer to use?
a) Sign language
b) Electrolarynx
c) Tracheoesophageal speech
d) Esophageal speech
e) Other:____________________________________

3. How many times, post-surgery, have you avoided water-related activities because of your laryngectomy?
a) 1-3 times
b) 3-5 times
c) 5-10 times
d) 10+ times
e) Never

4. What types of water-related activities would you enjoy participating in? Circle all that apply.
a) Fishing
b) Kayaking/canoeing/boating
c) Water skiing/tubing
d) Swimming
e) Snorkeling
f) Surfing/windsurfing
g) Parasailing
h) Other:______________________________________

5. Before your laryngectomy did you participate in water-related activities?
a) Yes
b) No

5a. If you answered yes, which sports did you participate in? Circle all that apply.
a) Fishing
b) Kayaking/canoeing/boating
c) Water skiing/tubing
d) Swimming
e) Snorkeling
f) Surfing/windsurfing
g) Parasailing
h) Other:_______________________________________

5b. How often did you participate in these activities per year? Circle all that apply.
a) 1 time
b) 2-4 times
c) 4-8 times
d) 8+ times
e) Never

5c. What was the average duration of the water activity? Circle all the apply.
a) 5-10 mins
b) 30 mins - 1 hour
c) 2-3 hours
d) 3+ hours
e) Other:____________________

6. Which is your dominant hand?
a) Left

b) Right

7. How likely would you be to use an assistive device to prevent water from accidentally entering the airway during participation in water-related activities?

a) Very likely
b) Likely
c) Not likely
d) Highly unlikely
e) Not sure

8. What is your intended use of the device?

a) Extreme water sports (e.g. Water skiing, snorkeling, scuba diving, etc.)
b) Relaxation water sports (e.g. Boating, fishing, walking on the beach, etc.)
c) Moderate water sports (e.g. swimming, kayaking, etc.)
d) I would not use the device

9. On a scale from 1-10, rate the ease with which you can breathe during moderate exertion (for example, when walking up stairs). (1 being very easy to breathe, and 10 being very difficult)

1 2 3 4 5 6. 7 8 9 10

10. Do you currently suffer from any breathing disorders?

a) Yes
b) No

11. How often would you anticipate taking the device on and off while participating in a water-related activity?

a) Always
b) Occasionally
c) Never

12. How much would you be willing to pay for an assistive device that protects the airway from water ingestion while participating in water-related activities?

a) Under $20
b) $20-$40
c) $40-$60
d) $60-$100
e) $100+

14. What do you think is the ideal travel size of an assistive device that protects the airway from water ingestion while participating in water-related activities?

a) Pocket Size
b) Backpack Size
c) Duffle bag size

13. Do you have any feedback/suggestions for the current prototype?

14. Are there any functions of features we haven't thought of that you think would be helpful or beneficial to be include on the device?
