# Supplementary material for: An automatic water-occluding device to enable laryngectomee participation in water activities
Source: PLoS One. 2021 Sep 13;16(9):e0257463. doi: 10.1371/journal.pone.0257463 (PMC8437266; doi:10.1371/journal.pone.0257463)
Supplement: Attachment — Submitted filename: Response_to_Reviewers_FINALdocx.pdf [file pone.0257463.s002.pdf]

Response to Reviewers
PONE-D-21-14821
"An automatic water-occluding device to enable laryngectomee participation in water activities"

We would like to thank the Associate Editor and the reviewers for their detailed and thoughtful comments. Below, we have addressed each reviewer's comments on a point-by-point basis. Reviewer comments are numbered, and presented in bold face text below. Our responses are posted in normal-face text. Changes to the manuscript are quoted using italics. In addition, a supplemental file has been included that highlights all changes to the manuscript. We hope this manuscript addresses the concerns of the reviewers.

Editor Comments:

1. **Thank you for your submission. This is an interesting paper about a potentially helpful device in laryngectomee patients. However, I agree with the reviewers that this study doe not prove safety. Publishing it as such may put patient's in harm's way and could be dangerous. This needs to be revised to be a proof of concept paper and there needs to be a warning that this study does not prove safety or recommend the use of the device until further investigation is carried out and FDA approval (or similar) is passed.**

Response:
These stipulations and warnings have been added to the manuscript. Please see the response to Reviewer 1, comments 2 and 4, below.

Reviewer #1:

1. **In their manuscript, the authors describe their novel device designed to allow laryngectomy patients the ability to engage in water activities.**

   There are some unnecessary commercial items listed around line 47.

Response:
The list of commercially-available stoma filtration products have been removed.

2. **The largest issue is the ethics of this study. The authors do not differentiate the risk of the various water sports (ie fishing from a dock vs paddleboarding or kayaking).**

Response:
Regarding assessing the risk of specific activities, it is not possible to quantitatively determine how risk may vary from one activity to another, or how an individual's tolerance of risk may influence their decision to participate in a given activity. To address this, and acknowledge that risks and risk tolerance may vary, the second-to last paragraph in the introduction has been updated to clearly identify that the approach for designing the current device was to develop a device that will protect someone if they fall into the water, as opposed to an individual that desires to participate in more extreme activities. It now reads:

"The potentially fatal consequences associated with these recreational water activities often results in laryngectomees eschewing them, despite pre-surgical participation/enjoyment, and/or desires to do so. Note, because risk varies by activity, the current approach focuses on providing protection when participating in activities where risk arises due to slipping and falling into the water (e.g., fishing, canoeing, boating, etc.), as opposed to more extreme activities such as jet-skiing, water-skiing, etc., where more robust protection would be needed."

3. **The integrity of the "waterproof" adhesive is not addressed.**

Response:
It has been clarified that the integrity of the stoma adhesive was not explicitly investigated. A new paragraph has been added to the section on Device Evaluation, which in part, reads:

"For example, the integrity of the waterproof adhesive that connects the stoma attachment to the skin of the neck was not explicitly tested. While the adhesive is specifically designed to be waterproof, the effectiveness of the adhesive when coupled to the device remains to be evaluated."

4. **This may be better suited as a proof of concept manuscript with explicit discussion that is not meant for actual patient use at this time.**

Response:
We agree with the reviewer that it is necessary to clearly identify that the current objective is an evaluation of a proof-of-concept, and that the device should not be considered appropriate or suitable for use. To clarify this point, the following text has been added to the end of the first paragraph of the section Device Design:

"It is emphasized that, as presented, the STORKEL is not recommended for current use as it has not received FDA approval. Furthermore, while the design approach of the device is evaluated, device safety is not proven in the current work. Consequently, the device should not be considered a suitable solution to prevent ingress of water through the stoma during participation in water activities until the appropriate approval and certification has been completed."

In addition, the wording in the abstract has been modified to identify that the current approach is a "*proof-of-concept*."

The new paragraph in the section on Device Evaluation includes a discussion on complications that could arise as testing was not performed with laryngectomees (see Reviewer 2, comment 1) and the following text has also been added:

"These potential complications highlight the need to consider the current device a proof-of-concept solution, and also identify possible challenges to be addressed in the future."

Finally, this has been emphasized again in the last sentence of the Conclusions, which now reads:

"It is emphasized that because the device does not have FDA approval, and the effectiveness of it when in use has not been proven, it is not currently suitable for personal use."

Reviewer #2:

1. **A well written manuscript about a new device for larygectomees' patients. As device was only tested in normal people, it remains untested in real patients and there may be unforeseen issues. I suggest to add these into the discussion. What are the unforeseen circumstances that may arise and what measures can be instituted to overcome them?**

Response:
As the editor and other reviewers identified, the device testing that was carried out in the current manuscript should only be considered "proof-of-concept", and does not prove efficacy or safety. As such, clarification regarding the risk of participating in different activities, as well as the type of protection the device was designed to address, has been added in the introduction as was discussed in response to Reviewer 1, point 2, above. Text was also added to the section on Device Design, clarifying that the current device does not prove safety (see the response to Reviewer 1, point 4, above).

In addition, the discussion in the Device Evaluation section has been expanded to identify potential unforeseen circumstances that may influence device performance in laryngectomees, and which were not explicitly investigated herein. A new paragraph has been added, which reads:

"Note that proof-of-concept evaluation of the device was not performed with laryngectomees. This may introduce additional circumstances that have not been explicitly addressed herein. For example, the integrity of the waterproof adhesive that connects the stoma attachment to the skin of the neck was not explicitly tested. While the adhesive is specifically designed to be waterproof, the effectiveness of the adhesive when coupled to the device remains to be evaluated. This is especially pertinent to laryngectomees who may suffer from skin degradation and localized sensitivity/inflammation due to radiation treatments for head and neck cancer. Similarly, varying morphometries of the stoma opening may influence fit, comfort and function of the stoma attachment. In addition, flow resistances through the device were determined to be suitable for normal breathing conditions. However, laryngectomees often have co-morbidities that influence pulmonary function. The influence of reduced lung capacity on the comfort and effectiveness of the device also remains to be evaluated. These potential complications highlight the need to consider the current device a proof-of-concept solution, and also identify possible challenges to be addressed in the future."

<u>Reviewer #3:</u>

1. **In reference to Line [8] of the Introduction, bilateral vocal fold paralysis and intractable aspiration are not causes of laryngeal dysfunction, but results. Cancer, trauma, or neurological insult are common etiologies of laryngeal impairment.**

<u>Response:</u>
The sentences have been changed to read:

"This typically occurs as a result of some underlying pathology. If the ability of the larynx to protect the airway becomes compromised, as may occur from laryngeal cancer \cite{Maddox12}, bilateral vocal fold paralysis \cite{Li17}, or intractable aspiration \cite{Eisele89}, surgical intervention via laryngotracheal separation \cite{Eisele89}, tracheoesophageal diversion \cite{Eisele89}, or a total laryngectomy \cite{Asai72} may be necessary."

2. **In line [15], the authors go on to state that the primary function of the larynx is speech production. Many would argue that the primary function is airway protection, which would support the premise for the device.**

<u>Response:</u>
Then sentence has been updated to read "*Because one of the functions of the larynx …*"

3. **The design and thus the name of the device is a bit confusing. SToma-snORKEL (STORKEL) implies a breathing device, not an arresting device. When the airway is submerged in water, the airway is closed on multiple levels, e.g., lips close, vocal folds adduct, diaphragm and lungs stop expanding, until the airway (typically the nose or mouth) has surfaced. For laryngectomees, the problem is twofold. First, the airway of a laryngectomee is redirected to the tracheostoma, and the vocal folds are no longer present to provide protection. Second, the stoma is positioned distally in the neck, which will often remain submerged when an individual is in deep water, unless they can float on their back and allow the stoma to rise above the surface.**

<u>Response:</u>
The STORKEL is both an arresting device that prevents incursion of water if the device is completely submerged, but also a breathing device that allows a laryngectomee to breathe (via the float valve) when the stoma is below the waterline, but the top of the STORKEL (attached to the head) is above the water.

This is analogous to a snorkeler putting their face below the water, where a snorkel allows them to breathe. Analogously, a laryngectomee, with their stoma below the water line is able to breathe with the assistance of the proposed device. Hence the name Stoma-snORKEL

(STORKEL).

4. **Based on that premise, one can see why a "snorkel" type device would be advantageous, to enable respiration, should the stoma remain submerged for an extended period of time. This concept could be highlighted, especially in the event that a life preserver may not provide enough buoyancy to keep the stoma above the waterline.**

Response:
While the device was designed to enable both protection of the airway during initial submersion, and continued breathing capability after resurfacing, this was not clearly explained in the objective statement. The last paragraph of the introduction has been modified to include this additional capability, and now reads:

"… the objective of this work is to design and evaluate a proof-of-concept stoma-occlusion/breathing device that can be used to protect the airway of laryngectomees during unanticipated submersion in water, while allowing the individual to breathe after resurfacing should their stoma be below the waterline. This scenario is …"

5. **While the occluding feature of the float valve was intensively assessed, the occlusion at the level of the stoma was not evaluated. If the main purpose of the device is to protect the stoma, then this should be assessed. Individuals who have undergone total laryngectomy often experience suboptimal baseplate seals due to sensitive skin and stoma landscape. Individuals who use a TEP experience additional challenges of excess pressure exerted on the baseplate when they occlude their stomas to generate TEP speech, thus often "break the seal." A broken seal while using this device would be life threatening. This could be easily tested on several laryngectomees (with IRB approval) by just having them speak and cough with the device on, and assessing if the seal stays intact.**

Response:
The reviewer is correct in that the seal at the stoma is an important consideration. This issue was also noted by Reviewer 1, comment 3. Unfortunately, despite requesting the IRB to allow subjects to try on the device for this exact purpose, it was denied due to concerns that it could inadvertently obstruct breathing, posing a serious threat.

Consequently, while subject specific testing was not possible, a discussion identifying this shortcoming, and the potential complications that could arise with securing the stoma attachment to a laryngectomee , have been added to the last paragraph of the Section Device Evaluation. It reads, in part:

“Note that proof-of-concept evaluation of the device was not performed with laryngectomees. This may introduce additional circumstances that have not been explicitly addressed herein. For example, the integrity of the waterproof adhesive that connects the stoma attachment to the skin of the neck was not explicitly tested. While the adhesive is specifically designed to be waterproof, the effectiveness of the adhesive when coupled to the device remains to be evaluated. This is especially pertinent to laryngectomees who may suffer from skin degradation and localized sensitivity/inflammation due to radiation treatments for head and neck cancer. Similarly, varying morphometries of the stoma opening may influence fit, comfort, and function of the stoma attachment…”

6. **The additional limitations and additional questions for future testing and design modification are suggested. In addition, did the authors present their design to the original laryngectomees who completed the design survey?**

Response:
Based on similar comments by Reviewer 2 comment 1, the last paragraph of the Device Evaluation section has addressed potential complications such as the aforementioned stoma fit, as well as how the device will work with individuals who have impaired breathing capacity. The latter half of the closing paragraph reads:

“*In addition, flow resistances through the device were determined to be suitable for normal breathing conditions. However, laryngectomees often have co-morbidities that influence pulmonary function. The influence of reduced lung capacity on the comfort and effectiveness of the device also remains to be evaluated. These potential complications highlight the need to consider the current device a proof-of-concept solution, and also identify possible challenges to be addressed in the future.”*

The authors did present their design to the laryngectomees following completion, and it was favorably received. However, because IRB approval was not requested to garner official feedback, it could not be included as part of the manuscript.

7. **It is commendable that the needs of individuals who have undergone total laryngectomy have caught the attention of mechanical engineers. Collaborating with clinicians in the field of head and neck surgery, and speech-language pathology may all the more expedite the effort.**

Response:
Thank you. Collaborations are ongoing.

Journal Requirements:
When submitting your revision, we need you to address these additional requirements.

1. **Please ensure that your manuscript meets PLOS ONE's style requirements, including those for file naming. The PLOS ONE style templates can be found at https://journals.plos.org/plosone/s/file?id=wjVg/PLOSOne_formatting_sample_main_body.pdf and https://journals.plos.org/plosone/s/file?id=ba62/PLOSOne_formatting_sample_title_authors_affiliations.pdf**

Response:
The manuscript has been compiled using the PLOS ONE Latex style file.

2. **Please provide additional details regarding participant consent. In the ethics statement in the Methods and online submission information, please ensure that you have specified whether consent was informed.**

Response:
The ethics statement has been added to the Design Objectives Section, including that the consent was informed.

3. **We note that the grant information you provided in the 'Funding Information' and 'Financial Disclosure' sections do not match. When you resubmit, please ensure that you provide the correct grant numbers for the awards you received for your study in the 'Funding Information' section.**

Response:
The discrepancy has been corrected.

4. **Please include your full ethics statement in the 'Methods' section of your manuscript file. In your statement, please include the full name of the IRB or ethics committee who approved or waived your study, as well as whether or not you obtained informed written or verbal consent. If consent was waived for your study, please include this information in your statement as well.**

Response:
The ethics statement has been added to the Design Objectives Section, including that the consent was informed.

5. **We note that Figures 1, 3, 4 and 5 in your submission contain copyrighted images. All PLOS content is published under the Creative Commons Attribution License (CC BY 4.0), which means that the manuscript, images, and Supporting Information files will be freely available online, and any third party is permitted to access, download, copy, distribute, and use these materials in any way, even commercially, with proper attribution. For more information, see our copyright guidelines: http://journals.plos.org/plosone/s/licenses-and-copyright.**

   We require you to either (1) present written permission from the copyright holder to publish these figures specifically under the CC BY 4.0 license, or (2) remove the figures from your submission:

   a. You may seek permission from the original copyright holder of igures 1, 3, 4 and 5 to publish the content specifically under the CC BY 4.0 license.

   We recommend that you contact the original copyright holder with the Content Permission Form (http://journals.plos.org/plosone/s/file?id=7c09/content-permission-form.pdf) and the following text:
   "I request permission for the open-access journal PLOS ONE to publish XXX under the Creative Commons Attribution License (CCAL) CC BY 4.0 (http://creativecommons.org/licenses/by/4.0/). Please be aware that this license allows unrestricted use and distribution, even commercially, by third parties. Please reply and provide explicit written permission to publish XXX under a CC BY license and complete the attached form."

   Please upload the completed Content Permission Form or other proof of granted permissions as an "Other" file with your submission.

   In the figure caption of the copyrighted figure, please include the following text: "Reprinted from [ref] under a CC BY license, with permission from [name of publisher], original copyright [original copyright year]."

   b. If you are unable to obtain permission from the original copyright holder to publish these figures under the CC BY 4.0 license or if the copyright holder's requirements are incompatible with the CC BY 4.0 license, please either i) remove the figure or ii) supply a replacement figure that complies with the CC BY 4.0 license. Please check copyright information on all replacement figures and update the figure caption with source information. If applicable, please specify in the figure caption text when a figure is

similar but not identical to the original image and is therefore for illustrative purposes only.

Response:
It is unclear why Figures 1, 3, 4, and 5 were deemed to be protected by copyright. They are all author-generated figures that have not been published elsewhere. As such, they should not require copyright permission.

6. **Please include captions for your Supporting Information files at the end of your manuscript, and update any in-text citations to match accordingly. Please see our Supporting Information guidelines for more information: http://journals.plos.org/plosone/s/supporting-information.**

Response:
A caption has been added, and a reference was added to the manuscript.
